# Is behavioural activation effective in the treatment of depression in young people? A systematic review and meta‐analysis

**DOI:** 10.1111/papt.12121

**Published:** 2017-03-16

**Authors:** Lucy Tindall, Antonina Mikocka‐Walus, Dean McMillan, Barry Wright, Catherine Hewitt, Samantha Gascoyne

**Affiliations:** ^1^ Mental Health and Addiction Research Group Department of Health Sciences University of York UK; ^2^ School of Psychology Deakin University Burwood Victoria Australia; ^3^ Child Oriented Mental Health Intervention Centre (COMIC) University of York UK; ^4^ York Trials Unit Department of Health Sciences University of York UK

**Keywords:** Adolescents, behavioural interventions, depression, meta‐analysis, systematic review

## Abstract

**Purpose:**

Depression is currently the leading cause of illness and disability in young people. Evidence suggests that behavioural activation (BA) is an effective treatment for depression in adults but less research focuses on its application with young people. This review therefore examined whether BA is effective in the treatment of depression in young people.

**Methods:**

A systematic review (International Prospective Register of Systematic Reviews reference: CRD42015020453), following Preferred Reporting Items for Systematic Reviews and Meta‐Analyses guidelines, was conducted to examine studies that had explored behavioural interventions for young people with depression. The electronic databases searched included the Cochrane Library, EMBASE, MEDLINE, CINAHL Plus, PsychINFO, and Scopus. A meta‐analysis employing a generic inverse variance, random‐effects model was conducted on the included randomized controlled trials (RCTs) to examine whether there were overall effects of BA on the Children's Depression Rating Scale – Revised.

**Results:**

Ten studies met inclusion criteria: three RCTs and seven within‐participant designs (total *n* = 170). The review showed that BA may be effective in the treatment of depression in young people. The Cochrane risk of bias tool and the Moncrieff scale used to assess the quality of the included studies revealed a variety of limitations within each.

**Conclusions:**

Despite demonstrating that BA may be effective in the treatment of depression in young people, the review indicated a number of methodological problems in the included studies meaning that the results and conclusions should be treated with caution. Furthermore, the paucity of studies in this area highlights the need for further research.

**Practitioner points:**

- Currently BA is included within National Institute for Health and Clinical Excellence (NICE, 2009) guidelines as an evidence‐based treatment for depression in adults with extensive research supporting its effectiveness. It is important to investigate whether it may also be effective in treating young people.
- Included studies reported reductions in depression scores across a range of measures following BA.
- BA may be an effective treatment of depression in young people.

## Background

By the year 2030, depression will be the leading cause of disease burden globally; it is already the leading cause of illness and disability in young people (World Health Organization [WHO, 2013, 2014]). In a large meta‐analysis (Costello, Erkanli, & Angold, 2006), the overall prevalence rates of depression were suggested to be 2.8% for children under 13 years of age and 5.7% for those aged between 13 and 18 years. It is important that young people experiencing depressive episodes are identified early and receive effective treatment to reduce negative symptoms and improve mood. Such treatments may assist young people to deal with the impact of their depression (i.e., on their family, social, and academic functioning) and reduce the likelihood or impact of future episodes (Birmaher & Brent, 2007).

However, despite high rates of depressive disorders, few young people seek help (O'Dea, Calear, & Perry, 2015; Rickwood, Deane, Wilson, & Ciarrochi, 2005). This limited help‐seeking may be influenced by factors associated with treatments including stigma (Gulliver, Griffiths, & Christensen, 2010; Rickwood *et al*., 2005), negative attitudes about help‐seeking (Rickwood *et al*., 2005), accessibility (Gulliver *et al*., 2010), and young people's reluctance to engage one‐to‐one with a therapist (Rickwood, 2010). Of those who do seek help, few receive it from specialist mental health services (Richardson, Stallard, & Velleman,^2010^), often as a result of limited clinician capacity and therapy availability (Roberts, 2013).

Increasing activity can be an important component in reducing depressive thoughts and feelings (Lejuez, Hopko, Le Page, Hopko, & McNeil, 2001). Behavioural activation (BA), a type of talking therapy focused on increasing adaptive activities, can be defined as a structured, brief psychotherapeutic approach that aims to (1) increase engagement in adaptive activities (which often are those associated with the experience of pleasure or mastery), (2) decrease engagement in activities that maintain depression or increase risk for depression, and (3) solve problems that limit access to reward or that maintain or increase aversive control’ (Dimidijian, Barrera, Martell, Munoz, & Lewisohn, 2011, p. 4).

According to Dimidijian *et al*. (2011), BA is defined by its reliance on behavioural principles with a specific focus on behaviour change. Several terms have been used to describe this type of treatment. In this study, the term ‘BA’ is used to encompass all therapies based upon this broad behavioural approach to the treatment of depression regardless of the specific terms used to describe the intervention.

Multiple studies have demonstrated the effectiveness of face‐to‐face BA in the treatment of depression in adults. Ekers, Richards, and Gilbody (2008) in a meta‐analysis of 17 studies found BA to be significantly superior to control conditions, brief psychotherapy and supportive therapy and comparable to CBT in its effectiveness for ameliorating symptoms of depression. Cuijpers, Van Straten, and Wamerdam (2007) found BA to be as effective as cognitive therapy, and Mazzuchelli, Kane, and Rees (2009) found a large effect size in favour of BA over controls. A systematic review (Chartier & Provencher, 2013) of studies (*n* = 21) comparing the efficacy of BA in treating depression to other psychotherapeutic and pharmacological interventions found BA to be as effective as other psychotherapies including cognitive behavioural therapy (CBT). This also provides some evidence that BA may be effective when delivered in a low intensity form (e.g., guided self‐help).

Although treatment recommendations and guidelines for individuals experiencing depression differ between adults and young people, given the extensive research supporting the use of BA with adults it is important to investigate whether it may also be effective in treating young people.

There has been less research on the use of BA with children and young people. Research in this area has generally been in the form of case series with small sample sizes, for example Chu, Colognori, Weissman, and Bannon (2009; *n* = 5); Wallis, Roeger, Milan, Walmsley, and Alison (2012; *n* = 5). Both of these reviews provided support for the use of BA in the treatment of young people with depression and/or anxiety, finding high treatment satisfaction and clinical benefits including symptom reductions. Similar results were obtained in a pilot, uncontrolled study of the use of BA for treating depressed young people in rural Australia (Jacob, Keeley, Ritschel, & Craighead, 2013), with all participants (*n* = 5) showing reduced levels of depressive symptoms between baseline and completion (at 6 months).

Given the paucity of research in this area and the lack of any published systematic review, an examination of BA for use with children and young people is required and timely (Chartier & Provencher, 2013). Young people experiencing depression may be treated more effectively using computerized therapies which have increased availability and accessibility (Stallard, Velleman, & Richardson, 2010), less stigma and are presented in a format attractive to many young people compared to traditional face‐to‐face therapies. Thus far, much research has focused on the delivery of CBT in this form (e.g., Abeles *et al*., 2009; Spence, Holmes, March, & Lipp, 2006; Spence *et al*., 2008; Wright *et al*., 2014), with computerized CBT (CCBT) representing an alternative form of therapy delivery that can be administered at a lower cost than traditional treatments (Merry *et al*., 2012). Although adult research has also often focused upon CCBT for depression, research into computerized BA has started to emerge. In a review by Spates *et al*. (2016), five web‐based BA interventions were identified all of which have demonstrated relative success in initial pilot trials.

Research is therefore required to establish whether BA in a computerized format has been used with young people experiencing depression also and, if so, whether it is an effective treatment.

This review sought to investigate (1) whether BA is effective in the treatment of depression in young people and, if so, (2) whether it can be effectively delivered in a computerized form.

## Methods

This review was completed with reference to the guidelines reported in the Preferred Reporting Items for Systematic Reviews and Meta‐Analyses (PRISMA) statement (Liberati *et al*., 2009). The review protocol was registered on the International Prospective Register of Systematic Reviews (PROSPERO), an online database for systematic review protocols (reference: CRD42015020453).

### Information sources and study identification

The following electronic databases were searched between July and August 2015: Cochrane Library, EMBASE, MEDLINE, CINAHL Plus (EBSCO), PsychINFO, Scopus, and the ISCRTN registry. To cover peer review and grey literature sources, the Health Management Information Consortium, NHS evidence, Open Grey, the Networked Digital Library of Theses and Dissertations, Web of Science Conference Proceedings, and ZETOC were searched with the search simplified accordingly. The reference lists of all included studies were examined and forward citation searching carried out in Google Scholar. No restrictions on publication status or language were imposed.

Titles and abstracts were screened independently by the primary researcher alongside a second reviewer to enhance the reliability of included studies. If any disagreements occurred between the two researchers, the two met to discuss these. In the event that a decision could not be reached between the two, a third researcher was consulted and asked to screen the disagreed paper(s) and make an overall decision regarding selection.

The search strategy used was based on three main constructs: *behavioural interventions* (including BA, behavioural therapy, behavioural interventions, self‐monitoring, and activity scheduling), *depression* (including depressive disorder, depressive, depression, and depressed) and *young people* (including adolescents, children, teen, youth, juvenile, pre‐pubescent, and student). See Appendix for the search strategy used.

### Eligibility criteria

The population of interest was young people aged 18 years and below. Studies employing a population that crossed the age of 18 were included if a minimum of 90% of the sample was under 18. Trial participants had to be experiencing depression or depressive symptoms as established by a validated screening measure or diagnosis based on a structured clinical interview conducted to internationally recognized standards (e.g., International Classification of Disease, Diagnostic Statistical Manual).

For inclusion, interventions had to be based upon either operant conditioning principles or comprise techniques fundamental to behavioural treatments of depression (activity scheduling, self‐monitoring, goal setting). Interventions based on third‐wave CBT principles (e.g., acceptance and commitment therapy) were excluded.

No restrictions were placed on comparator or control group types to avoid excluding any relevant studies reporting on BA. Studies that did not employ a control group were also included.

The primary outcome measure was levels of depression/depressive symptoms as measured by validated assessments. Assessments could include self‐report measures and clinician or researcher administrated ratings. Additional outcomes included levels of anxiety symptoms (measured by validated assessments), cost‐effectiveness, quality of life, and school attendance.

Randomized controlled trial (RCT) and pre‐/post‐study designs presenting relevant outcomes in a useable format were included in the review.

No restrictions were placed on intervention duration, delivery settings (e.g., community, health care, educational), or delivery mode (e.g., computerized, face‐to‐face), the timings of the measurement of the outcome measures nor upon sample sizes or sampling methodologies.

### Data extraction

Information extracted (using a pre‐piloted proforma) from included studies comprised study characteristics (study name, author(s), year of publication/production (if unpublished), study location, and setting), study design, study populations (basic demographics of participants, depression diagnosis methods), intervention details and comparators (intervention type, comparator, duration of the intervention, number of sessions), and relevant outcome data for effect size calculations (depression severity, unit of measurement).

### Quality assessments

The methodological quality of RCTs was formally assessed using the Cochrane risk of bias tool (Higgins *et al*., 2011), a general tool used to assess risk in any RCT and the Moncrieff scale (Moncrieff, Churchill, Drummon, & McGuire, 2001), specifically designed to assess the quality of controlled studies examining interventions for depressive and non‐psychotic symptoms. The Cochrane risk of bias tool categorises risk as ‘high’, ‘low’, or ‘unclear’ (where insufficient information is supplied to assess level of risk), whilst the Moncrieff scale awards scores (0–2) based on a study's success at addressing 23 risk items with higher scores representing higher levels of quality.

The inclusion of both scales allowed comparisons to be made between a general quality assessment tool and one specifically designed for use within the proposed field. The Moncrieff scale was also used to assess the quality of within‐participant design studies.

### Data synthesis and statistical analysis

A summary of the outcome measures of all included studies is provided alongside a forest plot providing a graphical display of the study outcomes of the RCTs. Owing to the limited number of RCTs, only one meta‐analysis was conducted for the Children's Depression Rating Scale – Revised (CDRS‐R; Poznanski & Mokros, 1996). Studies were pooled using the generic inverse variance method with a random‐effects model. All analyses were undertaken in stata version 13 (Stata Corporation, 2013). Statistical heterogeneity was assessed using the *I* ^2^ statistic with a value of 25% being regarded as low, 50% as moderate, and 75% as high (Higgins, Thompson, Deeks, & Altman, 2003). Publication bias could not be examined due to insufficient numbers of included studies.

## Results

### Literature search results

**Figure 1:**
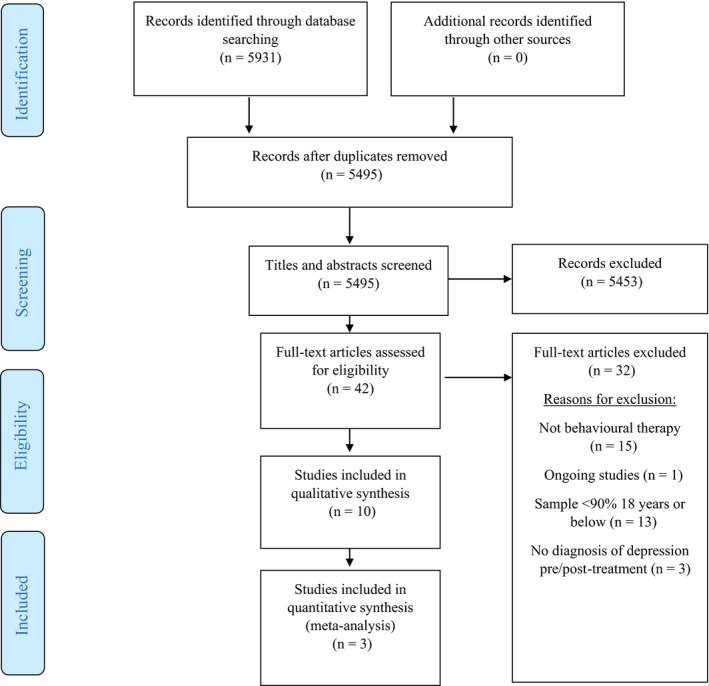
Preferred Reporting Items for Systematic Reviews and Meta‐Analyses diagram.

The original search, including grey literature searching, identified 5,931 potentially relevant records of which 5,495 remained after duplicates (*n* = 436) were removed. No additional studies were found from forward citation searches. The screening of titles and abstracts was undertaken by the primary and secondary reviewer who identified 42 full‐text articles for assessment. No disagreements as to studies eligible for inclusion occurred. After relevance checking was independently conducted, 10 papers were deemed eligible for inclusion (Figure 1). Reasons for exclusion of the other 32 included <90% of the sample aged 18 or below (*n* = 13; Armento, 2011; Armento, McNulty, & Hopko, 2012; Gawrysiak, Nicholas, & Hopko, 2009; Harmon, Nelson, & Hayes, 1980; Levin *et al*., 2010; Ly *et al*., 2014; Mohammadi, Birashk, & Gharaie, 2013; Moradveisi, Huibers, Renner, Arasteh, & Arntz, 2013; Parker *et al*., 2011; Proudfoot *et al*., 2013; Shaw, 1977; Takagaki *et al*., 2013; Velayudhan, Gayatridevi, & Bhattacharjee, 2010), treatments not meeting the criteria to be regarded as BA (*n* = 15; Bilek & Ehrenreich‐May, 2012; Brent, Kolko, Birmaher, Baugher, & Bridge, 1999; Brent *et al*., 1997, 1998; Chu, Hoffman, Johns, Reyes‐Portillo, & Hansford, 2015; Dundon, 2010; Esposito, 2005; Ettelson, 2003; Kauer *et al*., 2011; Landback *et al*., 2009; Merry, McDowell, Wild, Bir, & Cunliffe, 2004; Nystedt, 1977; Reid *et al*., 2011; Sobowale *et al*., 2013; Van Voorhees *et al*., 2008), being an ongoing study with currently unreported results (*n* = 1; C. E. W. Kitchen, D. Ekers, P. A. Tiffin, & S. Lewis, Personal communication) or examining a sample which had not received a diagnosis of depression at baseline (*n* = 3; Davidson *et al*., 2014; Pass, Brisco, & Reynolds, 2015; Reynolds, Macpherson, Tull, Baruch, & Lejuez, 2011).

### Characteristics of included studies

Of the ten studies that met inclusion criteria for the review, three were RCTs (Chu *et al*., 2016; McCauley *et al*., 2015; Stark, 1985) and seven used a within‐participant design (Chu *et al*., 2009; Douleh, 2013; Jacob *et al*., 2013; Riley & Gaynor, 2014; Ritschel, Ramirez, Jones, & Craighead, 2011; Wallis *et al*., 2012; Weersing, Gonzalez, Campo, & Lucas, 2008). The studies varied greatly regarding sample sizes, settings, participant demographics, and the interventions and comparators (if applicable) employed. Overall, 170 participants were included across the ten studies ranging in age from 8 to 18 years.

The majority of studies (*n* = 7) measured depression using the Children's CDRS‐R (Poznanski & Mokros, 1996). The CDRS‐R is the most widely used measure of child and adolescent depression severity and demonstrates high inter‐rater reliability (Pozanski and Mokros: α = .92), good 2‐week test–retest reliability (.80), and good to excellent internal consistency (α = .74–.92) within this context (Mayes, Bernstein, Haley, Kennard, & Emslie, 2010).

Five of the ten studies also collected outcome data relating to anxiety, whilst only one reported on quality of life. None of the other secondary outcomes of interest, cost‐effectiveness, and school attendance was reported in any of the studies. Table 1 provides a summary of the descriptive characteristics of the included studies.

**Table 1:** A descriptive summary of the characteristics of included studies (*n* = 10)

Study	Subjects	Setting	Study design	Intervention	Comparator	Outcome measures	Data collection points
Chu *et al*. (2016) USA	35 young people (10 males, 25 females), aged 12–14 (mean: 12.03) with a current clinical principal diagnosis of either a unipolar depression disorder or an anxiety disorder based on CDRS‐R or ADIS‐IV	One public middle school	RCT	10 one‐hour sessions of group behavioural activation therapy (GBAT) Delivered face‐to‐face (1 clinical psychologist, 4 graduate students, 2 school counsellors) *N* = 21 (intention to treat – 2 lost to follow‐up)	Wait list control *N* = 14 (intention to treat – 1 lost to follow‐up)	CDRS‐R CES‐D ADIS‐IV SCARED	Pre‐treatment Post‐treatment 4‐month follow‐up
McCauley *et al*. (2015) USA	60 young people (38 females, 22 males), aged 12–18 (mean: 14.9) with a depressive disorder based on the K‐SADS diagnostic interview	One hospital‐based mental health clinic	RCT	14 sessions of adolescent behavioural activation program (A‐BAP) Delivered face‐to‐face (2 doctoral students, 1 social worker) *N* = 35 (intention to treat – 8 lost to follow‐up)	Up to 14 sessions of a face‐to‐face delivered, evidence‐based practice for depression *N* = 25 (intention to treat – 9 lost to follow‐up)	K‐SADS diagnostic interview CDRS‐R SMFQ MASC	Pre‐treatment Post‐treatment 6‐month follow‐up 12‐month follow‐up
Stark (1985) USA	29 young people (16 males and 13 females) aged 9–12 (mean: 11.2) with a depressive disorder based on the CDI (≥16)	One elementary school	RCT	12 45‐minute sessions of behaviour therapy delivered over 5 weeks Delivered face‐to‐face (1 study therapist, 1 clinical psychologist) *N* = 10, 9 included in analysis	Twelve 45 minute sessions of face‐to‐face delivered self‐control therapy over 5 weeks or wait list control *N* = self‐control therapy: 9 (all included in analysis, wait list: 9 (all included in analysis)	CDI CDS CDRS‐R RCMAS	Pre‐treatment Post‐treatment 8‐week follow‐up
Riley and Gaynor (2014) USA	11 participants (9 males and 2 females) aged 8–12 (mean: 9.8) with a depressive disorder as based upon scores on the CDRS‐R (≥12) and CDI (≥40)	3 elementary schools, 1 middle school	Within‐participant design	3 sessions of face‐to‐face delivered non‐directive therapy (NDT) only over 3 weeks: *N* = 4, all included in the analysis or 3 sessions of face‐to‐face delivered non‐directive therapy (NDT) over 3 weeks: Followed by 9 sessions of behaviour therapy (BT)	None	CDRS‐R CDI FQOLS	Pre‐treatment Post‐treatment (both groups) 2‐month follow‐up
Douleh (2013) USA	14 participants (8 males and 6 females) aged 14–18 (mean: 15.7) with a depressive disorder based upon scores on the CDRS‐R (≥45)	2 High schools	Within‐participant design	1–4 sessions of Motivational Interviewing (MI) over 4 weeks Delivered face‐to‐face (study therapist) *N* = 14 (2 lost to follow‐up) or 1–4 sessions of MI and 1–4 sessions of Fun activities (FA) Delivered face‐to‐face (study therapist) *N* = 7 (3 lost to follow‐up) or 1–4 sessions of MI, 1–4 sessions of FA and 1–4 sessions of values‐based behavioural activation (VBBA) Delivered face‐to‐face (study therapist) *N* = 1 (1 included in analysis)	None	CDRS‐R BDI‐II MINI‐KID HRQOL (measure not specified)	A1: Pre‐treatment A2: Post‐MI (4 weeks after pre‐treatment) A3: Post‐FA (10 weeks after pre‐treatment) A4: Post‐VBBA (16 weeks after pre‐treatment) A5: 20 weeks after pre‐treatment
Ritschel *et al*. (2011) USA	6 young people (3 males and 3 females) aged 14–17 with a depressive disorder based on the K‐SADS or CDRS‐R (≥45)	Outpatient adolescent mood clinic	Within‐participant design	22 sessions of behavioural activation delivered over 18 weeks Delivered face‐to‐face (2 doctoral level staff, 1 graduate student) *N* = 6 (all included in analysis)	None	KSADS CDRS‐R BDI‐II	Pre‐treatment Post‐treatment
Chu *et al*. (2009) USA	5 young people (2 males and 3 females) aged 12–14 with a current clinical principal diagnosis of either a unipolar depression disorder or an anxiety disorder based on CES‐D (≥15) or ADIS‐IV (no cut‐offs specified)	One public middle school	Within‐participant design	13 sessions of group behavioural activation therapy (GBAT) delivered over 13 weeks Delivered face‐to‐face (mental health specialists) *N* = 5 (all included in analysis)	None	CES‐D ADIS‐IV MASC	Pre‐treatment Post‐treatment (13 weeks)
Wallis *et al*. (2012) Australia	5 participants (all female) aged 14–15 with a diagnosis of a depressive disorder based upon the CES‐D (no cut‐offs specified)	Local mental health service	Within‐participant design	10 sessions of behavioural activation delivered over 10 weeks Delivered face‐to‐face (2 social workers) and work books *N* = 5 (all included in analysis)	None	BDI‐II	Pre‐treatment 2 weeks 3 weeks 6 weeks Completion (10 weeks)
Weersing *et al*. (2008) USA	2 participants (1 male and 1 female) aged 13 and 17 with a depressive disorder based on CDI (≥13) or an anxiety disorder based on the SCARED (≥25)	Primary care practice	Within‐participant design	8 30‐minute sessions of Integrated brief behavioural therapy for anxiety and depression delivered over 12 weeks Delivered face‐to‐face (mental health specialists) *N* = 2 (all included in analysis)	None	CDI K‐SADS SCARED	Pre‐treatment Post‐treatment (12 weeks) 24‐week follow‐up
Jacob *et al*. (2013) USA	3 participants (2 males and 1 female) aged 14–17 with a depressive disorder based on K‐SADS, CDRS‐R (≥45) and BDI‐II (≥14)	Community mental health clinics	Within‐participant design	14–17 sessions of behavioural activation (adapted for low‐income, African American adolescents) delivered over 6 months Delivered face‐to‐face (3 study therapists) *N* = 3 (all included in analysis)	None	KSADS CDRS‐R BDI‐II	Pre‐treatment At each session (BDI‐II) Week 9 (CDRS‐R) Post‐treatment (6 months)

Notes

*Depression measures*: CDRS‐R, Children's Depression Rating Scale – Revised (Poznanski & Mokros, 1996); SMFQ, Short Mood and Feelings Questionnaire (Angold, Costello, Messer, & Pickles, 1995); CES‐D, Center for Epidemiologic Studies Depression Scale (Radloff, 1977); CDI, Children's Depression Inventory (Kovacs, 2011); CDS, Children's Depression Scale (Reynolds, 1980); BDI‐II, Beck Depression Inventory (Beck, Steer, & Brown, 1996); K‐SADS, Kiddie Schedule for Affective Disorders (Kaufman *et al*., 1997); MINI‐KID, Mini International Neuropsychiatric Interview for Children and Adolescents (Sheehan *et al*., 2010). *Anxiety measures*: MASC, Multi‐Dimensional Anxiety Scale for Children (March, Parker, Sullivan, Stallings, & Conners, 1997); SCARED, Screen for Anxiety Related Emotional Disorders (Birmaher *et al*., 1999); RCMAS, Revised Children's Manifest Anxiety Scale (Reynolds & Richmond, 1978); ADIS‐IV, Anxiety Disorders Interview Schedule for DSM‐IV Child Interview (Silverman & Albano, 1996). *Quality of life measures:* FQOLS, The Family Quality of Life Scale–Family Interactions Subscale (Hoffman, Marquis, Poston, Summers, & Turnbull, 2006). RCT, randomized controlled trials.

### Quality assessments

#### Randomized controlled trials

On the Cochrane risk of bias tool (Table 2), all three RCTs demonstrated low risk regarding the reporting of other sources of bias, incomplete outcome data, and blinding outcome assessors. Level of bias was unclear where no, or insufficient, information regarding a particular domain (allocation concealment (Chu *et al*., 2016; Stark, 1985), random sequence generation; Stark, 1985) was provided. In addition, with no pre‐published protocols, it was hard to determine whether all pre‐specified outcomes had been reported in each of the three RCTs and therefore the level of bias due to selective reporting was unclear. As the three RCTs involved the delivery of BA neither participants nor personnel could be blinded to treatment allocation. However, a lack of blinding meant that all three RCTs demonstrated a high risk of bias on this domain.

**Table 2:** Cochrane risk of bias and included randomized controlled trials (*n* = 3)

Domain	McCauley *et al*. (2015)	Chu *et al*. (2016)	Stark (1985)
Random sequence generation	+	+	?
Allocation concealment	+	?	?
Selective reporting	?	?	?
Other sources of bias	+	+	+
Blinding participants and personnel	−	−	−
Blinding (outcome assessment)	+	+	+
Incomplete outcome data	+	+	+

Notes

‘+’ low risk of bias; ‘−’ high risk of bias; ‘?’ unknown risk of bias.

On the Moncrieff scale (Table 3), all three RCTs attained maximum quality scores for method of allocation, providing clear descriptions of treatment, using clear diagnostic criteria and the recording of exclusion criteria. However, on a number of the domains examined, although bias was minimized, it was still present (e.g., in conducting appropriate statistical analyses and assessment of compliance to treatments). High risk of bias was reported where studies provided no or insufficient information about power calculations, concealment of allocation (Chu *et al*., 2016; Stark, 1985), sample sizes, and declarations of interest (Stark, 1985). Only the study by Chu *et al*. (2016) attained a maximum score for reporting side effects, with neither of the other two RCTs discussing this.

**Table 3:** Moncrieff scale – included randomized controlled trials (RCTs) (*n* = 3)

Domain	RCTs		
	McCauley *et al*. (2015)	Chu *et al*. (2016)	Stark (1985)
Objections and specifications, main outcomes *a priori*	1	2	2
Adequate sample size	2	2	0
Appropriate duration of trial and follow‐up	2	2	1
Power calculations	2	0	0
Method of allocation	2	2	2
Concealment of allocation	2	0	0
Clear description of treatments	2	2	2
Blinding of subjects	N/A	N/A	N/A
Sources of subjects/representative sample	1	2	2
Use of diagnostic criteria	2	2	2
Record of exclusion criteria	2	2	2
Description of sample demographics	1	2	2
Blinding of assessor	1	2	1
Assessment of compliance with treatments	1	1	1
Details of side effects	0	2	0
Record of number and reasons for withdrawal	2	1	2
Outcome measures described clearly	2	2	2
Information on comparability and adjustment for difference in analysis	2	1	2
Inclusion of all subjects in analysis (ITT)	2	2	2
Presentation of results with inclusion of data for re‐analysis of main outcomes	2	2	2
Appropriate statistical analysis	1	1	1
Conclusions justified	2	2	2
Declarations of interest	2	2	0
Total	36	36	30

Notes

Maximum total score is 56; higher scores denote lower bias.

#### Within‐participant designs

Only one domain on the Moncrieff scale (Table 4) attained a maximum quality score on all seven included studies. This related to the outcome measures used with all studies using validated and reliable instruments. However, none of the studies conducted power calculations, blinded assessors, employed adequate sample sizes, or reported any information about the side effects of treatment. As a result, every study was awarded zero suggesting the presence of bias on each of these domains. For the remainder of bias domains on the Moncrieff scale, most studies attained a score of one or two suggesting measures had been taken to minimize the level of bias. However, a number of studies attained scores of zero in relation to the following: bias related to statistical analyses (Chu *et al*., 2009; Jacob *et al*., 2013; Wallis *et al*., 2012; Weersing *et al*., 2008), not providing information on comparability and adjustment for differences in analyses (Chu *et al*., 2009; Jacob *et al*., 2013; Ritschel *et al*., 2011; Wallis *et al*., 2012; Weersing *et al*., 2008), not reporting compliance and treatment adherence (Wallis *et al*., 2012; Weersing *et al*., 2008), and having a limited follow‐up period (Riley & Gaynor, 2014; Wallis *et al*., 2012).

**Table 4:** Moncrieff scale – within‐participant design studies (*n* = 7)

Domain	Within‐participant designs						
	Chu *et al*. (2009)	Jacob *et al*. (2013)	Ritschel *et al*. (2011)	Wallis *et al*. (2012)	Weersing *et al*. (2008)	Douleh (2013)	Riley and Gaynor (2014)
Objections and specifications, main outcomes *a priori*	2	1	1	1	1	2	2
Adequate sample size	0	0	0	0	0	0	0
Appropriate duration of trial and follow‐up	1	1	1	0	2	1	0
Power calculations	0	0	0	0	0	0	0
Method of allocation	N/A	N/A	N/A	N/A	N/A	N/A	N/A
Concealment of allocation	N/A	N/A	N/A	N/A	N/A	N/A	N/A
Clear description of treatments	2	2	2	0	2	2	2
Blinding of subjects	N/A	N/A	N/A	N/A	N/A	N/A	N/A
Sources of subjects/representative sample	1	1	1	1	1	1	1
Use of diagnostic criteria	1	2	2	1	1	2	2
Record of exclusion criteria	1	2	2	1	1	2	2
Description of sample demographics	2	2	2	0	2	2	2
Blinding of assessor	0	0	0	0	0	0	0
Assessment of compliance with treatments	2	2	2	0	0	1	2
Details of side effects	0	0	0	0	0	0	0
Record of number and reasons for withdrawal	2	2	2	2	0	1	1
Outcome measures described clearly	2	2	2	2	2	2	2
Information on comparability and adjustment for difference in analysis	0	0	0	0	0	1	1
Inclusion of all subjects in analysis (ITT)	2	2	2	0	0	2	2
Presentation of results with inclusion of data for re‐analysis of main outcomes	1	1	1	0	0	1	1
Appropriate statistical analysis	0	0	1	0	0	1	1
Conclusions justified	2	2	2	0	2	1	2
Declarations of interest	0	0	0	0	0	0	2
Total	21	22	23	8	14	22	25

Notes

Maximum total score is 56; higher scores denote lower bias.

Through not employing a randomized methodology, two items on the Moncrieff scale (methods of allocation and concealment of allocation) were not applicable to the within‐participant designs.

### BA effectiveness and depression

The effectiveness of BA is reported separately for each of the two types of study designs included in the review (RCTs, within‐participant designs). Owing to the limited number of RCTs included, only one meta‐analysis could be conducted. However, a forest plot depicting depression outcomes on all measures employed within each of the three RCTs is provided.

#### Randomized controlled trials

**Figure 2:**
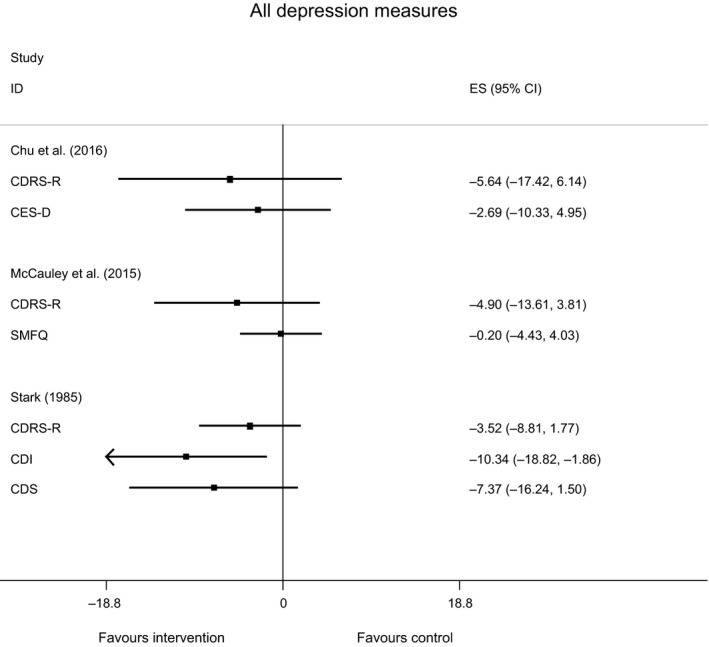
Forest plot of all depression measures across included randomized controlled trials (*n* = 3).

Two of the included RCTs measured depression outcomes using two continuous measures of depression (Chu *et al*., 2016: CDRS‐R, Center for Epidemiologic Studies Depression Scale [CES‐D]; McCauley *et al*., 2015: CDRS‐R, Short Mood and Feelings Questionnaire [SMFQ]), whilst the third reported on three (Stark, 1985: CDRS‐R, Children's Depression Inventory [CDI], Children's Depression Scale [CDS]). A forest plot was produced to provide a graphical display of these study outcomes (Figure 2). During data extraction, it was noted that in one of the studies (Chu *et al*., 2016), there was a baseline imbalance in depression severity scores (intervention 42.57 [*SE* 5.08], wait list 46.00 [*SE* 3.95]) and an error in the reporting of the standard error for the depression severity scores post‐intervention (wait list *SE* = 0; intervention *SE* = 5.36). Rather than exclude this study from the analysis, it was decided to use the reported estimates and standard errors from the adjusted models (including adjustments for baseline scores) to ensure inappropriately large differences were not attributed to the intervention.

All three RCTs measured depression using the CDRS‐R and demonstrated reductions in depression scores using this measure. McCauley *et al*. (2015) reported that mean CDRS‐R scores between pre‐treatment and end of treatment reduced from 57.6 (*SD*: 11.8) to 40.18 (*SD*: 13.9) for those receiving BA in comparison with a reduction from 57.8 (*SD*: 8.3) to 45.05 (*SD*: 14.2) for those receiving treatment as usual. At end of treatment, 76% of participants randomized to BA scored forty or below on the CDRS‐R, indicating a depression diagnosis to be either ‘possible’ or ‘unlikely’ in comparison with 42% of those receiving treatment as usual. These pre‐treatment to end‐of‐treatment outcomes fell within the 95% confidence interval suggesting reliability in the change scores. In the study by Chu *et al*. (2016), CDRS‐R depression scores reduced from 42.6 (*SD*: 5.08) to 37.67 (*SD*: 5.36) from pre‐treatment to post‐treatment in the BA group, whilst scores increased from 46.0 (*SD*: 3.95; pre‐wait list) to 57.0 (*SD*: 0.00; post‐wait list) in the control group. Owing to the small sample size employed within this study, statistical analyses were not performed, and therefore, the significance of these results cannot be inferred. Finally, Stark (1985) reported reductions in mean CDRS‐R depression scores for those receiving BA to be from 33.50 (*SD*: 10.27) at pre‐treatment to 24.02 (*SD*: 6.01) at end of treatment and then to 24.28 (*SD*: 4.68) at follow‐up. For those receiving self‐control therapy, mean CDRS‐R scores reduced from 37.22 (*SD*: 8.36) at pre‐treatment to 22.90 (*SD*: 4.36) at end of treatment and to 20.69 (*SD*: 3.45) at follow‐up. Reductions in the wait list group reduced from 27.57 (*SD*: 3.51) at pre‐treatment to 27.24 (*SD*: 5.74) at end of treatment and to 22.60 (*SD*: 5.03) at follow‐up. However, the results of an ANCOVA test demonstrated that the difference between groups on the CDRS‐R at end of treatment was not significant (*p* < .30).

**Figure 3:**
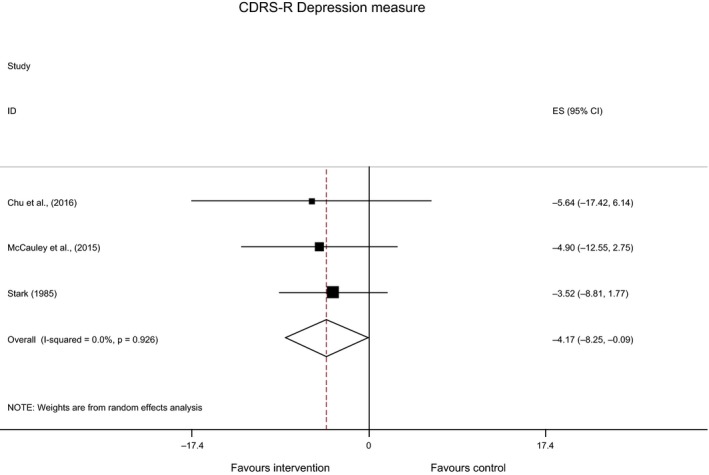
Random‐effects meta‐analysis of the CDRS‐R across included randomized controlled trials (*n* = 3).

As the included RCTs were judged to be sufficiently similar, a meta‐analysis was conducted for the CDRS‐R. The effect of BA on CDRS‐R depression scores was moderate with a pooled mean difference of −4.17 (95% CI: −8.25, −0.09; Figure 3). This demonstrates a statistically significant difference in CDRS‐R scores in favour of BA. The *I* ^2^ statistic was 0% (*p *=* *.926) suggesting no statistical heterogeneity was present (Higgins *et al*., 2003).

In relation to the other depression measures, in the study by Stark (1985), mean depression scores as measured by the CDI reduced in the BA group from 22.40 (*SD*: 8.47) at pre‐treatment to 9.11 (*SD*: 8.32) at end of treatment and to 7.43 (*SD*: 7.23) at follow‐up. In the self‐control therapy, mean CDI scores reduced from 21.60 (*SD*: 5.48) at pre‐treatment to 8.09 (*SD*: 6.65) at end of treatment and to 5.36 (*SD*: 5.04) at follow‐up, whilst scores in the wait list control group reduced from 20.00 (*SD*: 10.71) at pre‐treatment to 19.45 (*SD*: 10.31) at end of treatment and then to 7.40 (*SD*: 5.68) at follow‐up. The results of an ANCOVA test demonstrated that these between‐group differences were statistically significant (*p* < .01) at the end of treatment. Thus, those receiving treatment had a greater reduction in depression scores, at this point than those in the wait list control group.

In the same study (Stark, 1985), mean depression scores as measured by the CDS also reduced in the BA group from 71.10 (*SD*: 10.38) at pre‐treatment to 55.24 (*SD*: 12.18) at end of treatment and to 50.03 (*SD*: 13.23) at follow‐up. In the self‐control therapy group, CDS scores reduced from 72.40 (*SD*: 10.31) at pre‐treatment to 50.29 (*SD*: 8.63) at end of treatment and then further reduced to 46.46 (*SD*: 8.31) at follow‐up. For the wait list group, mean CDS scores reduced from 66.00 (*SD*: 18.80) at pre‐treatment to 62.61 (*SD*: 7.14) at end of treatment and then to 48.20 (*SD*: 13.29) at follow‐up. These differences, however, fell short of conventional levels of statistical significance (*p* < .07).

On the CES‐D in the study by Chu *et al*. (2016), rates of depression reduced in the BA group from 21.00 (*SD*: 2.15) to 16.38 (*SD*: 2.30) compared to a reduction from 20.22 (*SD*: 2.73) to 19.07 (*SD*: 3.15) in the wait list group. However, once again statistical tests were not performed due to a small sample size and lack of control.

The final continuous measure used within the three RCTs was the SMFQ. In the BA group, mean scores reduced from 16.1 (*SD*: 6.1) at pre‐treatment to 6.3 (*SD*: 7.4) at end of treatment in comparison with a reduction from 15.6 (*SD*: 6.2) at pre‐treatment to 6.5 (*SD*: 6.5) at end of treatment in the treatment as usual group. These differences in groups were, however, not significant (*p* = .53).

In addition to the continuous measures reported, McCauley *et al*. (2015) conducted the Kiddie Schedule for Affective Disorders (K‐SADS) diagnostic interview with participants. Results demonstrated that 77% of BA participants no longer met diagnostic criteria for depression at end of treatment compared to 25% in the treatment as usual group.

#### Within‐participant designs

In the within‐participant design studies, two studies (Douleh, 2013; Riley & Gaynor, 2014) employed a stepped‐care approach, whilst the remainder (Chu *et al*., 2009; Jacob *et al*., 2013; Ritschel *et al*., 2011; Wallis *et al*., 2012; Weersing *et al*., 2008) conducted case series studies.

In the study by Riley and Gaynor (2014), all participants received non‐directive therapy (NDT) and, if not demonstrating improved depression ratings, subsequently received BA. Of those who received BA, 57% demonstrated a clinically significant change on both the CDRS‐R and the CDI at the end of treatment. There were also significant differences on both measures from post‐NDT to post‐BA (CDRS‐R: *M* = 41.57 [11.79]: *Z* = −2.37, *p *=* *.02; CDI: *M* = 16.29 [10.24]: *Z* = −2.37, *p *=* *.02).

All participants in the study by Douleh (2013) received motivational interviewing (MI) followed by fun activity (FA) sessions if not demonstrating reductions in depression ratings and subsequently BA (values‐based behavioural activation) if depression was still evident after both MI and FA. Overall, only one participant in this study received BA. For this participant, depression scores on the CDRS‐R reduced from 53 at baseline to 31 post‐BA and 25 at follow‐up. Reductions on the Beck Depression Inventory (BDI‐II) were also reported, reducing from 31 at baseline to 14 post‐BA, and to 11 at follow‐up.

In all within‐participant designs employing a case series methodology, reductions in depression scores were evident following BA. Jacob *et al*. (2013) and Wallis *et al*. (2012) reported reduced scores on the BDI‐II for all participants from baseline to trial completion with the participants in the latter of these two studies attaining depressive scores in the ‘normal’ range on this measure. Jacob *et al*. (2013) also reported reductions in depressive scores on the CDRS‐R and the K‐SADS with 2/3 participants no longer meeting the criteria of a depressive disorder following BA. Similarly, Ritschel *et al*. (2011) reported significant reductions in depressive scores as measured by both the BDI‐II and the CDRS‐R with 66% of participants being in the ‘normal’ range following treatment completion and thus similar to a non‐clinical sample. In the study by Weersing *et al*. (2008), both participants demonstrated a decrease in depression scores on the CDI from baseline to six‐month follow‐up, whilst Chu *et al*. (2009) reported significant reductions in depression scores on the CES‐D for 2/5 participants. All reported results for the within‐participant design studies can be seen in Table 5.

**Table 5:** Within‐participant design studies table of results

Study	Measure	Pre‐treatment Mean (*SD*) *n*	Post‐treatment Mean (*SD*) *n*	Follow‐up Mean (*SD*) *n*
Riley and Gaynor (2014)	*CDRS‐R*	55.36 (12.36) *n* = 11	41.57 (11.79) *n* = 7	–
	*CDI*	22.73 (9.29) *n* = 11	16.29 (10.24) *n* = 7	–
	*FQOLS*	17.14 (6.04) *n* = 11	21.29 (7.68) *n* = 7	–
Douleh (2013)	*CDRS‐R*	58.79 (9.11) *n* = 14	31 *n* = 1	25 *n* = 1^a^
	*BDI‐II*	21(11.48) *n* = 14	14 *n* = 1	11 *n* = 1^a^
Ritschel *et al*. (2011)	*CDRS‐R*	57.67 (11.18) *n* = 6	27.67 (8.07) *n* = 5	N/A
	*BDI‐II*	28.00 (6.51) *n* = 6	6.00 (5.87) *n* = 5	N/A
Chu *et al*. (2009)	*CES‐D*	36.80 (6.22) *n* = 5	32.25 (14.39) *n* = 4	N/A
	*MASC*	51.40 (13.37) *n* = 5	50.00 (19.13) *n* = 4	N/A
Wallis *et al*. (2012)^b^	*BDI‐II*	25 (10.22) *n* = 5	12.2 (7.79) *n* = 5	N/A
Weersing *et al*. (2008)^b^	*CDI*	23 (2) *n* = 2	9.5 (1.5) *n* = 2	4 (2) *n* = 2^c^
	*SCARED*	32.5 (11.5) *n* = 2	13 (10) *n* = 2	8.5 (8.5) *n* = 2^c^
Jacob *et al*. (2013)	*CDRS‐R*	59.3 (13.6) *n* = 3	33.0 (19.1) *n* = 3	N/A
	*BDI‐II*	21.7 (4.1) *n* = 3	4.0 (2.0) *n* = 3	N/A

a Follow‐up at 20 weeks.

b Results not available therefore scores estimated from figures presented within the study papers, – incomplete data reported.

c Follow‐up at 24 weeks.

### BA effectiveness and other outcomes

Besides depression, this review also sought to examine the effectiveness of BA and several additional outcomes of interest. These were levels of anxiety symptoms, cost‐effectiveness, quality of life, and school attendance.

#### Randomized controlled trials

All three RCTs examined the effectiveness of BA in the treatment of anxiety. Chu *et al*. (2016) reported greater reductions in anxiety scores for those receiving BA (29.67 [*SD*: 2.23] to 21.05 [*SD*: 2.41]) compared to those in the wait list group (28.51 [*SD*: 3.36] to 26.93 [*SD*: 4.56]) from pre‐ to post‐treatment as measured by the Screen for Anxiety Related Emotional Disorders (SCARED). Once again owing to the small sample size employed within this study, statistical analyses were not performed, and therefore, the significance of these results cannot be inferred. Finally, using the Revised Children's Manifest Anxiety Scale, Stark (1985) reported statistically significant reductions in anxiety from pre‐ to post‐testing in those receiving either BA or self‐control therapy (*p* < .01) and no improvement for those in the wait list condition. Individuals who received self‐control therapy demonstrated the highest reductions in anxiety at post‐testing.

None of the included RCTs reported on the cost‐effectiveness of BA nor on its impact on quality of life or school attendance.

#### Within‐participant designs

Of the seven within‐participant design studies, two reported on the effectiveness of BA for treating anxiety (Chu *et al*., 2009; Weersing *et al*., 2008). Based on the SCARED, both participants in the study by Weersing *et al*. (2008) demonstrated reduced anxiety scores following BA. For one participant, anxiety scores reduced across all time points following treatment completion; however, for the other, anxiety scores had increased again by six‐month follow‐up. In the study by Chu *et al*. (2009), two of the five included participants had reduced anxiety scores, as measured by the MASC, following BA. For the remaining three, one saw an increase in their anxiety score, for one it remained the same, whilst the other withdrew from treatment and did not complete follow‐up measures.

Only one included study (Riley & Gaynor, 2014) examined BA and quality of life. Using the Family Quality of Life Scale–Family Interactions Subscale, a significant increase in quality of life was found by the conclusion of BA (*M* = 21.29 [7.68]: *Z* = −2.21, *p* = .03). None of the included within‐participant designs reported on the cost‐effectiveness of BA nor on its impact on school attendance.

### Computerized BA

Although the ten studies included within this review provided information regarding the effectiveness of BA for young people with depression, none of the studies delivered BA in a computerized form. Therefore, the second objective of this review: whether BA can be effectively delivered in a computerized form, could not be investigated. Furthermore, none of the studies reported on the impact of BA on school attendance or investigated the cost‐effectiveness of the treatment – the remaining secondary outcomes specified.

## Discussion

This systematic review aimed to assess the effectiveness of BA in the treatment of adolescents with depression and investigate whether BA delivered in a computerized form is effective within this treatment context.

Across all included studies, regardless of methodology, reductions in depression were evident following BA. At the individual level, several of the findings were statistically significant, and when the RCT studies were combined within the meta‐analysis, a statistically significant difference in CDRS‐R scores from pre‐ to post‐treatment was found in favour of BA.

The findings of this review provide some preliminary evidence that BA may be an effective treatment of depression in young people. Not only were reductions in depression scores reported following BA across studies, within the RCTs these reductions were greater in comparison with those randomized to a control group. These findings are similar to those previously reported in adult studies where BA has been found to be superior to control conditions (e.g., Ekers *et al*., 2008; Mazzuchelli *et al*., 2009).

Besides depression, several of the included studies examined the effectiveness of BA on two of the secondary outcome measures – anxiety and quality of life. Five of the included studies (Chu *et al*., 2016; McCauley *et al*., 2015; Stark, 1985; Chu *et al*., 2009; Weersing *et al*., 2008) measured anxiety scores with all reporting reductions following BA. In relation to quality of life, in the one study that reported on it (Riley & Gaynor, 2014), there was a significant increase found by the conclusion of BA. These findings provide preliminary evidence that BA may also be effective in reducing anxiety and increasing quality of life for young people experiencing depression.

The second aim of this systematic review was to investigate whether BA delivered in a computerized form is effective in the treatment of young people with depression. Despite research suggesting the effectiveness of computer‐delivered therapies for young people (e.g., Merry *et al*., 2012; Stallard *et al*., 2010), none of the ten included studies delivered BA in a computerized form, and therefore, this aim could not be addressed. In addition, none examined the impact of BA on school attendance or investigated the cost‐effectiveness of the treatment.

### Limitations

Although this review has generated important information relating to the effectiveness of BA in the treatment of depression in young people, a number of methodological limitations need to be considered when interpreting the findings.

Firstly, as only three RCTs were included within the review, only one meta‐analysis could be completed. Although a pooled mean difference could be calculated and supports the effectiveness of BA for the treatment of depression, the results need to be interpreted with caution. The discrepancies noted with one of the included RCTs (Chu *et al*., 2016) and the subsequent adjustment made may have impacted upon the accuracy of the results reported. In addition, through the inclusion of only three studies in the meta‐analysis, explorations of publication bias could not be conducted.

The methodological flaws identified in all included studies may also have impacted upon the results presented. For example, a number of studies (e.g., Chu *et al*., 2016; Stark, 1985) provided no information regarding allocation concealment which may have inflated the effect sizes in favour of positive results (Shulz & Grimes, 2002; Wood *et al*., 2008). As several studies (e.g., Chu *et al*., 2009; Jacob *et al*., 2013) did not conduct any statistical analyses, their findings could only be inferred. In addition, the sample sizes of the included studies were low with the maximum number recruited in any study being 60 (McCauley *et al*., 2015). It must be noted that this was the only study to employ power calculations. Therefore, it is unclear whether the other two RCTs or within‐participant design studies recruited sufficient numbers of participants to identify an intervention effect.

In two of the studies, a transdiagnostic approach was taken (Chu *et al*., 2009; Weersing *et al*., 2008) whereby BA was used to treat both depression and anxiety. Although there is often comorbidity between anxiety and depression and so it was added within this review as a secondary outcome, the primary focus was on the effectiveness of BA for treating depression. Thus, it may be hard to distinguish between the elements of BA effective for treating depression and those for anxiety within these studies as the two are reported collectively.

Finally, no information was supplied in any of the studies about delivery of BA in a computerized form nor on the additional outcomes under review (school attendance, cost‐effectiveness). These were deemed as important factors at inception of the review but unfortunately cannot be reported on.

### Conclusions

This review was conducted to examine the effectiveness of BA for treating young people with depression. Ten studies (three RCTs and seven within‐participant designs) met the inclusion criteria and were subsequently included. The results provided some initial evidence that BA may be an effective treatment of depression in young people.

A number of methodological constraints in the included studies mean that the results need to be interpreted with caution. Such constraints need to be addressed in any future research.

## Search strategy

1. Behavio* activation.ti,ab.
2. (behavio* adj2 (intervention* or therap* or treatment* or psychotherap* or psycho‐therap*)).ti,ab.
3. Behavior Therapy/
4. Cognitive Therapy/
5. self monitor*.ti,ab.
6. (Activit* adj3 (schedul* or plan* or arrang* or organis* or organiz*)).ti,ab.
7. 1 or 2 or 3 or 4 or 5 and 6
8. Depression/
9. exp Depressive Disorder/
10. Depression.ti,ab.
11. Depressive.ti,ab.
12. Depressed.ti,ab.
13. ((low or negative or decreas*) adj2 (mood* or affect)).ti,ab.
14. 8 or 9 or 10 or 11 or 12 or 13
15. 7 and 14
16. young people.ti,ab.
17. young person*.ti,ab.
18. (child* or schoolchild*).ti,ab.
19. teen*.ti,ab.
20. adoles*.ti,ab.
21. youth*.ti,ab.
22. student*.ti,ab.
23. juvenile*.ti,ab.
24. pre‐pubert*.ti,ab.
25. (pre‐pubert* or prepubert*).ti,ab.
26. (pre‐pubescen* or prepubescen*).ti,ab.
27. (pre‐teen* or preteen*).ti,ab.
28. (puberty or pubertal).ti,ab.
29. Child/
30. Adolescent/
31. 16 or 17 or 18 or 19 or 20 or 21 or 22 or 23 or 24 or 25 or 26 or 27 or 28 or 29 or 30
32. 7 and 14 and 31
33. exp animals/ not humans.sh.
34. 32 not 33
